# Questions on the Structure and Development of the Human Teeth

**Published:** 1876-01

**Authors:** 


*Questions on the Structure and Development of the Human Teeth*, for the use of dental students. By C. L. Ford, M. D., Prof. of Anatomy and Physiology in the University of Michigan. Publishers: Fisk & Douglas, Ann Arbor, 1876.

This pamphlet comprises an excellent series of questions on the alveolar process, blood vessels, nerves, and structure of the teeth, structure of bone, muscles of mastication, development of the different tissues, and comparative anatomy of the dental organs, together with questions on some collateral subjects, such as bones of the head, maxillary sinus, etc. It will prove a useful adjunct to the text books.

